# Cortical hemodynamic responses and deep learning models of emotional face processing in preschool children with autism spectrum disorder: a fNIRS study

**DOI:** 10.3389/fpsyt.2025.1703302

**Published:** 2026-01-16

**Authors:** Liping Qi, Jing-Wen Ni, Guijun Dong, Tao Sun, Jian-Wei Zhang

**Affiliations:** 1 School of Control Science and Engineering, Dalian University of Technology, Dalian, China; 2 School of Computer Science, Dalian University of Technology, Dalian, China; 3 Quzhou University, Quzhou, China

**Keywords:** preschool children, autism spectrum disorder, functional near-infrared spectroscopy (fNIRS), cerebral hemodynamic response, convolutional neural network-long short-term memory model

## Abstract

**Purpose:**

The purpose of the present study was to characterize cortical hemodynamic responses during emotional face processing in preschool children with autism spectrum disorder (ASD) using functional near-infrared spectroscopy (fNIRS), and to develop machine learning frameworks for emotion recognition based on these hemodynamic signals.

**Methods:**

Fifty-three ASD preschoolers (41 males, 12 females; aged 3–7 years, mean age 5.20 ± 1.23 years) were exposed to dynamic video and static image facial stimuli displaying angry, happy expressions, and neutral flowers, with their brain activity concurrently recorded using whole-brain fNIRS. A convolutional neural network-long short-term memory (CNN-LSTM) model was proposed to decode spatiotemporal neural patterns of angry/happy emotion recognition.

**Results:**

fNIRS analysis revealed significantly enhanced activation in bilateral dorsolateral prefrontal cortex (DLPFC) and frontal pole during dynamic versus static stimulus processing. Angry expressions elicited the most pronounced neural responses, engaging a distributed cortical areas involving DLPFC, ventrolateral prefrontal cortex, and primary visual areas. The CNN-LSTM architecture achieved 86.2% accuracy in dynamic angry/happy emotion classification.

**Conclusion:**

This study provides evidence of altered cortical hemodynamics during dynamic emotional facial processing and demonstrates the feasibility of CNN-LSTM models for the objective assessment of emotional facial processing potential in preschool children with ASD.

## Introduction

Autism Spectrum Disorder (ASD) is a complex neurodevelopmental condition characterized by deficits in social communication and restricted repetitive behaviors (1). Globally, its rising prevalence presents significant challenges for individuals with ASD and their families (2) (3). A hallmark of ASD is persistent difficulty in interpreting nonverbal cues, particularly facial expressions, which undermines social interaction across developmental stages (F. Y. N. 4). While facial expressions are fundamental to emotional communication, individuals with ASD often struggle to recognize them as communicative signals, leading to impaired social engagement. Compared to neurotypical peers, ASD children show lower emotion identification accuracy, compromising their social adaptability (5).

Notably, despite early childhood being a critical window for neuroplasticity (6), most research focuses on adolescent and adult ASD populations. The preschool period (3–7 years) represents a pivotal stage where targeted interventions may promote neural reorganization. This study addresses this gap by using functional near-infrared spectroscopy (fNIRS), a neuroimaging modality ideal for pediatric research. Unlike fMRI, fNIRS requires no prolonged immobility, a limitation that restricts fMRI use in young children, and offers superior temporal resolution (7). Its non-invasive, portable, and motion-tolerant design enables hemodynamic measurements in challenging populations, driving growing interest in using fNIRS to study neural mechanisms in young children with ASD (8, 9).

The experimental paradigm uses dynamic and static facial expressions to enhance ecological validity, addressing limitations of traditional static stimulus designs. Behavioral studies show dynamic stimuli improve emotion discrimination in ASD by enhancing biological motion perception, yielding higher recognition accuracy than static images (10). Most prior face recognition research in ASD has involved older individuals with verbal tasks (4), yet age moderates facial affect recognition, with the performance gap between ASD and neurotypical groups widening over development. In the present study, we used fNIRS to measure cortical responses to happy/angry facial expressions in 3–7-year-old preschool children with ASD.

Recent advances in artificial intelligence (AI) have facilitated computational emotion analysis (11), whereas brain imaging techniques enable noninvasive detection of neural signatures for emotion classification in individuals with ASD (12) (13). Neural activity offers higher specificity for emotional categories than behavioral measures, containing discriminative features for emotion recognition (14). The proliferation of deep learning (DL) algorithms has rendered automatic feature learning via backpropagation increasingly viable, streamlining workflows and mitigating computational overhead relative to conventional machine learning (ML) paradigms. Eastmond et al. reported in a systematic review that 26 out of 32 DL-driven approaches outperformed traditional ML models in fNIRS research (15). Within the DL landscape, CNNs, RNNs, and LSTMs are extensively adopted for fNIRS-based classification, each tailored to distinct signal characteristics (15). LSTMs are well-characterized for capturing temporal variability in sequential data, which is critical for fNIRS, as hemodynamic responses exhibit inherent time-varying dynamics. In contrast, CNNs excel at extracting local spatial features from multi-channel fNIRS data but lack capacity to model long-range inter-regional interactions or temporal dependencies (16). In the present study, we proposed a cascaded CNN-LSTM architecture, which could accommodate fNIRS’s spatio-temporal nature. Therefore, the purposes of the present study were to: (1) compare neural activation patterns between dynamic and static facial expressions using fNIRS hemodynamics; (2) identify emotion-specific neural signatures in preschoolers with ASD; and (3) develop a deep learning framework for emotion recognition from fNIRS data. By identifying neural correlates of facial emotion processing deficits and exploring the integration of fNIRS and machine learning, this study aims to advance objective assessment of emotion recognition for early ASD intervention—an unmet need in clinical psychiatry, where subjective evaluation tools remain the dominant approach. Notably, the objective assessment tools developed herein may facilitate early identification of emotion processing impairments in ASD, thereby enabling timely and tailored intervention strategies to improve long-term outcomes for affected children.

## Methods

**Figure 1:**
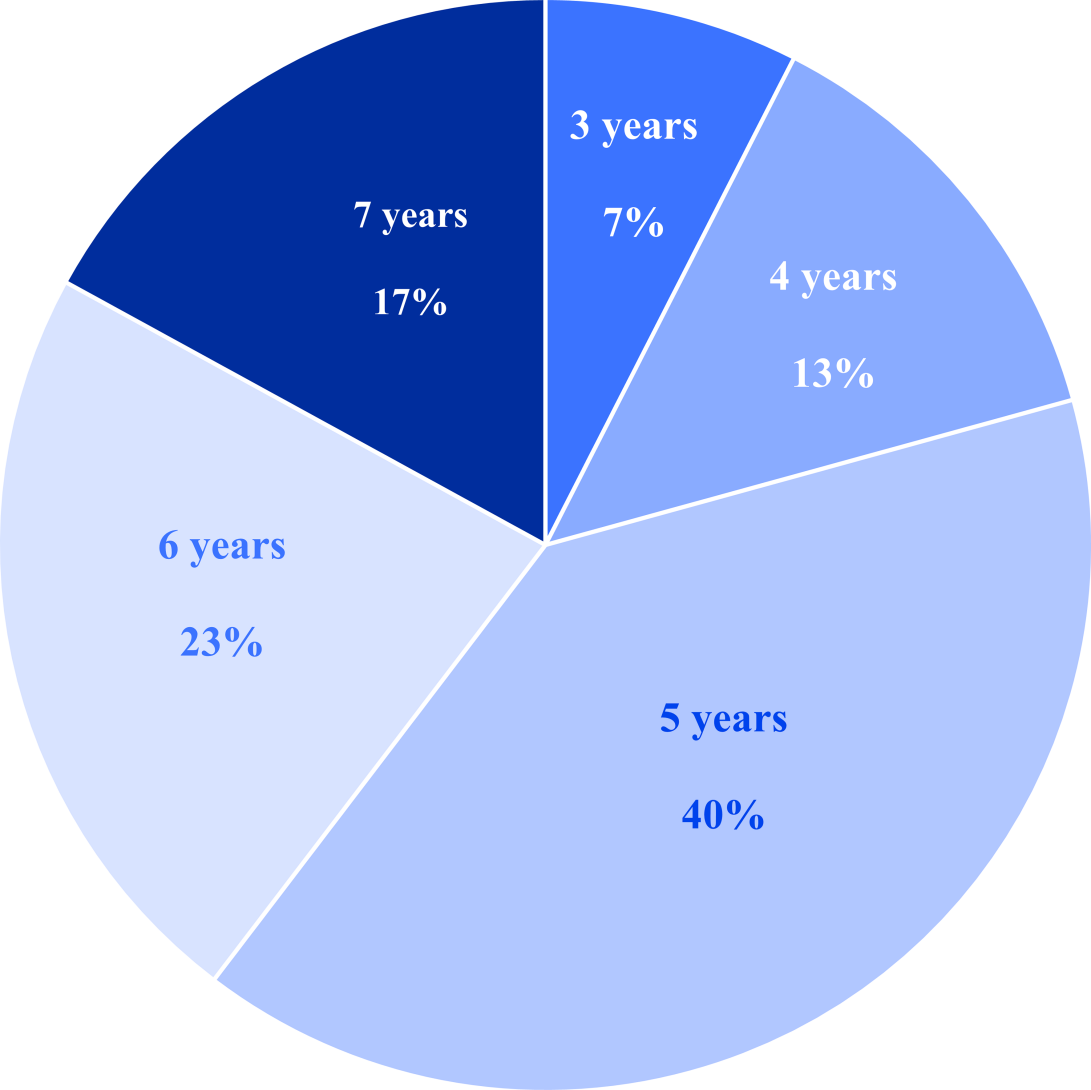
The age distribution of 53 preschoolers with ASD in the emotion recognition test.

A total of 53 children with autism spectrum disorder (41 males, 12 females; aged 3–7 years, mean age 5.20 ± 1.23 years) were recruited from Lixin Minkang Hospital, Shandong Province, China. Age distribution is presented in **Figure 1**. ASD diagnosis was confirmed using the Autism Diagnostic Observation Schedule (ADOS) and expert clinical assessment. Participants were excluded if they had a history of neurological/neurodevelopmental disorders (other than ASD for the clinical group) or an IQ below 65. Inclusion criteria required no prior antipsychotic medication use and typical intellectual functioning as assessed by the Chinese Wechsler Intelligence Scale for Children. All participants underwent resting-state and emotional face processing tasks. The study was approved by the hospital research ethics board and university ethics committee (ID: 2022027). Informed assent was obtained from the children, and written informed consent was obtained from their parents.

### Participants

### Procedure

**Figure 2:**
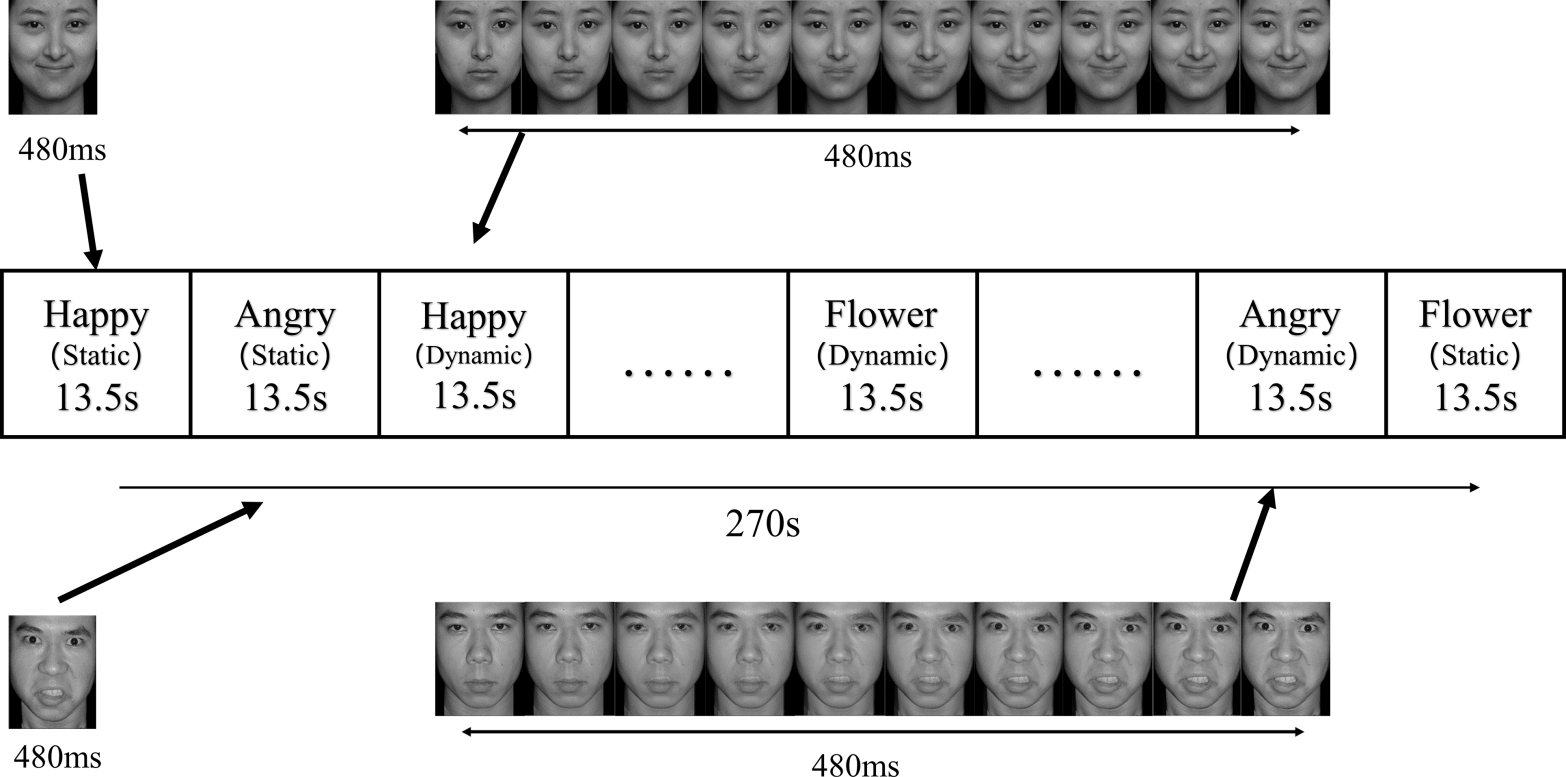
Emotional facial processing task. Dynamic (10-frame videos) and static (single-image final frames) emotional expressions (happy/angry) were presented for 480 ms each. The task used an ABXBAX randomized block design (A=dynamic, B=static, X=baseline) with four 13.5-second blocks per condition.

Stimuli consisted of grayscale images of happy/angry faces (8 males, 8 females) and flowers (control stimuli), presented in static or dynamic form (**Figure 2**). Happy and angry faces were selected because they are the two most commonly experienced emotions in young children, making them widely used in child-focused studies (17) (18). Faces were selected from the Chinese Affective Picture System (19), which includes validated expressions with > 80% recognition accuracy. Images were cropped using an oval mask to remove hair/ears/shoulders and set against a uniform light grey background. Control stimuli included static flower images and dynamic blooming sequences. Dynamic stimuli were generated using WinMorph software, morphing faces from neutral to smiling expressions over 10 frames. These were compiled into 480-ms videos at 50 fps, with the final frame repeated for 9 frames (24 frames total). Static stimuli displayed only the final frame for 480 ms. Stimuli were presented in an ABXBAX alternating block design (A=dynamic, B=static, X=baseline), with each condition comprising four 13.5 s blocks (20). Each block included 8 stimuli (1,500 ms inter-stimulus interval), preceded by a 1s white fixation cross. A star probe (1,500 ms) appeared randomly within blocks, prompting participants to press a key for attention maintenance. Baseline blocks (13.5 s, fixation cross) were interspersed, with 16-s fixation periods at the run’s start/end.

**Figure 3:**
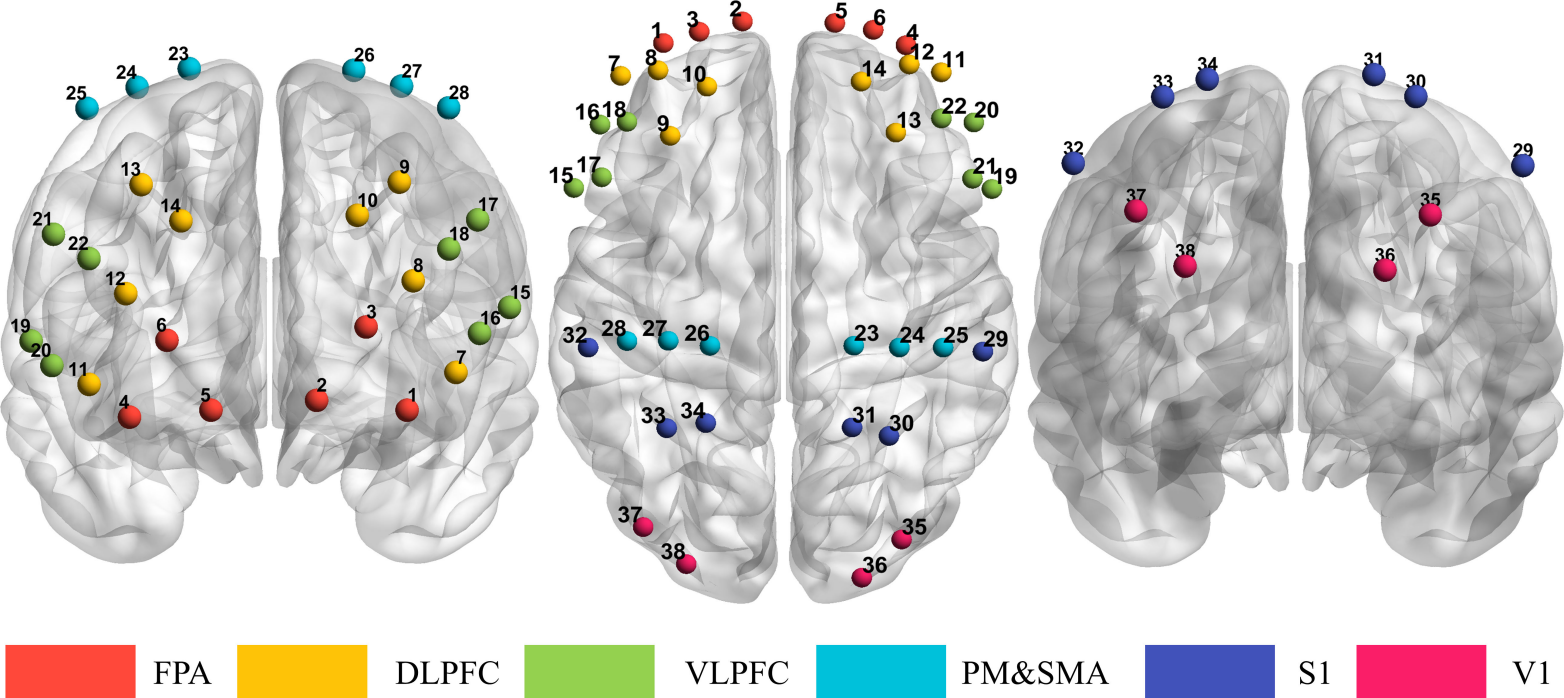
The configuration of fNIRS channels use in the present study. 20 emitters and 16 detectors arranged at an inter-probe distance of 3 cm, resulting in 38 channels per set. Estimated fNIRS channel locations are exhibited in MNI space. Six regions of interest in the prefrontal cortex and motor cortex are indicated with colors and channel numbers. FPA, frontopolar area; DLPFC, dorsolateral prefrontal cortex; VLPFC, ventrolateral prefrontal; PM&SMA, pre-motor/supplementary motor cortex; S1, primary somatosensory cortex; V1, primary visual cortex.

fNIRS signals were recorded using a multichannel system (Nirsmart; Huichuang Medical Equipment Co., Ltd., Beijing, China) during emotional face processing tasks. Two wavelengths of 730 and 850nm were used. Thirty-eight channels were configured with 20 light emitters and 16 detectors, set at a 3-cm inter-probe distance, as shown in **Figure 3**. A customized probe cap was designed to maintain sensor positioning, guided by the international 10–20 system: anterior sensors were placed around FP1/FP2, posterior sensors near PO7/PO8, left-lateral sensors around T3, and right-lateral sensors around T4. During probe placement, the acquisition software provided real-time signal quality evaluation and visualization for each channel. Regions of interest (ROIs) included the frontopolar area (FPA), dorsolateral prefrontal cortex (DLPFC), ventrolateral prefrontal cortex (VLPFC), pre-motor/supplementary motor cortex (PM&SMA), primary somatosensory cortex (S1), and primary visual cortex (V1) in both hemispheres. The sampling frequency was 10 Hz. To enhance compliance in children with ASD, each participant underwent a one-month pre-adaptation protocol: wearing a similar probe cap for 10 minutes daily prior to the study.

#### fNIRS signal processing

Optical density signals were converted into hemoglobin concentration changes using the modified Beer-Lambert law, with the differential pathlength factor adjusted based on participant age to account for age-dependent cerebral tissue properties. The SPM-fNIRS toolbox was used to analyze the fNIRS data (21, 22). Motion artifacts, a common challenge in fNIRS data due to head movements, were mitigated using a moving window and spline interpolation method. The moving window length was set to 1 second, the threshold factor to 3, and the smoothing factor to 5. Physiological noise such as cardiac pulsation and respiration was reduced using band-stop filters targeting 0.12–0.35 Hz and 0.7–2.0 Hz. Additionally, a discrete cosine transform-based high-pass filter suppressed very low-frequency drifts below 0.01 Hz, such as blood pressure fluctuations. Temporal autocorrelation in hemodynamic signals was addressed via pre-whitening, modeled as a first-order autoregressive process combined with white noise.

First-level analyses employed a general linear model (GLM) to fit hemodynamic responses, contrasting task periods with baseline intervals (fixation cross presentation). This GLM estimated channel-specific hemodynamic response parameters, deriving participant-specific effects of interest from channel-wise signal dynamics. Statistical parametric mapping (SPM) was used to generate individual-level brain maps highlighting task-induced hemodynamic changes, with the canonical hemodynamic response function (HRF) implemented in SPM. Group-level analysis utilized these maps in a two-way ANOVA with a 2×2 factorial design considering face categories (anger, happiness, and flowers), and stimulus states (dynamic and static). Family-wise error rate correction was applied through permutation testing with 10,000 iterations, and statistical significance was defined as *p*<0.05.

#### Emotion recognition modeling

To optimize deep learning model performance, fNIRS Signals were scaled to the [0, 1] range using the Min-max normalization formula. Physiological signal outliers were removed using a 3-standard-deviation threshold. Processed data from each participant were categorized into six task conditions based on emotional type (anger, happiness, neutral) and presentation mode (dynamic, static): dynamic anger, static anger, dynamic happiness, static happiness, dynamic neutral, and static neutral. Each condition included three 13.5-second blocks, with each yielding a task matrix of 38×405 (number of fNIRS channels × time steps). Furthermore, contrast-based differences were calculated for four pairs: dynamic anger vs. dynamic neutral, dynamic happiness vs. dynamic neutral, static anger vs. static neutral, and static happiness vs. static neutral. These contrast-based differences served as the training targets for the classifiers, which enhancing the model’s discriminative power by highlighting discrepancies in neural responses between emotional states and neutral (baseline) states. Subsequently, classification tasks for dynamic emotions and static emotions (anger vs. happiness) were trained separately. Labels were assigned based on whether the data groups included “anger” or “happiness” annotations (0=anger, 1=happiness), and a random training/test set split (4:1) was implemented.

**Figure 4:**
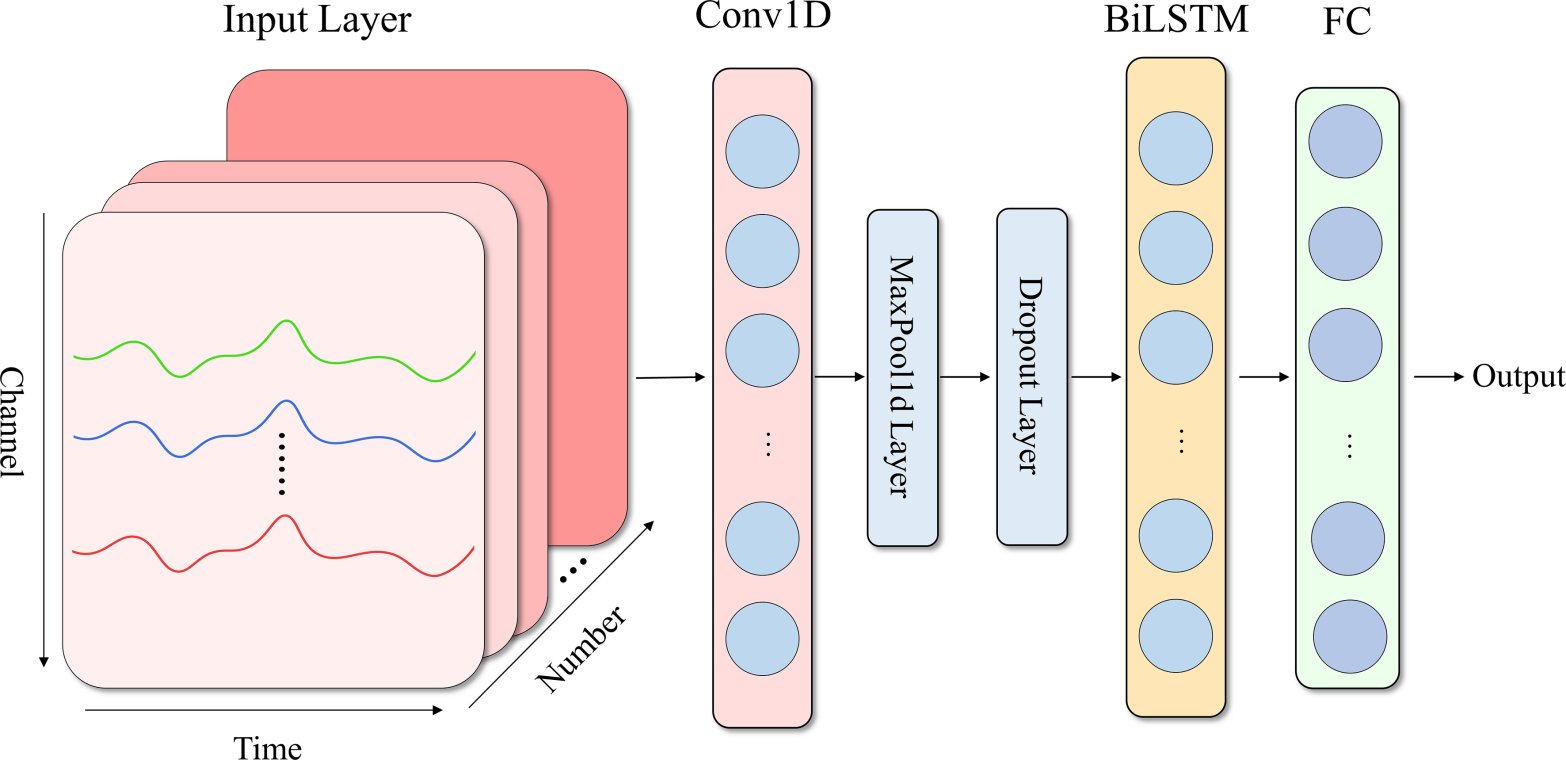
Framework of the proposed CNN-LSTM model.

A CNN-LSTM architecture was developed to decode emotional states from fNIRS contrast signals. fNIRS collects cerebral blood oxygenation changes via multiple channels placed on the scalp, resulting in data that contains both spatial and temporal information. Specifically, the proposed model first extracts spatial features through CNN layers and then employs LSTM layers to model temporal dynamics, forming a hierarchical feature learning framework. The architecture includes an input layer, Conv1D layer, Sigmoid layer, global max pooling layer, micro-probabilistic dropout layer, bidirectional LSTM layer, ReLU activation layer, and fully connected classification layer, as shown in **Figure 4**. The CNN component captures local spatial patterns from multi-channel signals, such as synergistic activation features across brain regions. The model converts the input data matrix (time steps, number of channels) into a (number of channels, time steps) matrix, enabling 1D-CNN kernels to slide along the channel dimension. This transformation focuses convolution operations on spatial correlations between channels rather than the temporal dimension, facilitating better learning of fNIRS spatial characteristics. 1×1 convolution kernels can independently extract features from each channel while preserving the spatial positional relationships between channels. Weight sharing is then used to learn collaborative patterns of adjacent channels, endowing the model with local spatial feature extraction capability. The LSTM component deciphers the dynamic evolution of hemodynamic responses over time, such as delayed neural activity under dynamic facial stimuli. The Adam algorithm is selected as the optimizer, and the learning rate was adjusted according to the complexity of the task. In order to deal with the high-dimensional sparse characteristics of fNIRS signals, global pooling was used to avoid excessive compression of key information. Additionally, bidirectional LSTM layers were introduced to strengthen the capture of contextual temporal correlations, which is crucial for analyzing asymmetric brain region activation patterns under dynamic stimuli. The structural parameters of the CNN-LSTM model are shown in **Table 1**.

**Table 1:** Structural parameters of the CNN-LSTM hybrid model.

Layer	Type	Parameters
1	Input	Input size=38
2	Conv1D	Kernel size=1,Stride=1,Padding=0
3	Act	Sigmoid
4	MaxPool1D	Kernel size=405,Padding=0,Dilation=1
5	Dropout	P=0.001
6	LSTM	Num_layers=1,Batch_first=True,Bidirectional=True
7	Act	ReLU
8	FC	Linear units=3(2)

The data is partitioned into training and test sets at a 4:1 ratio, with a fixed random seed to ensure consistency. In the training stage, the five-fold cross-validation was used to evaluate the generalization performance of the model. The optimal parameters of the validation set were saved in each round of training, and the final test results were averaged for five times to reduce the influence of random fluctuations.

## Results

**Figure 5:**
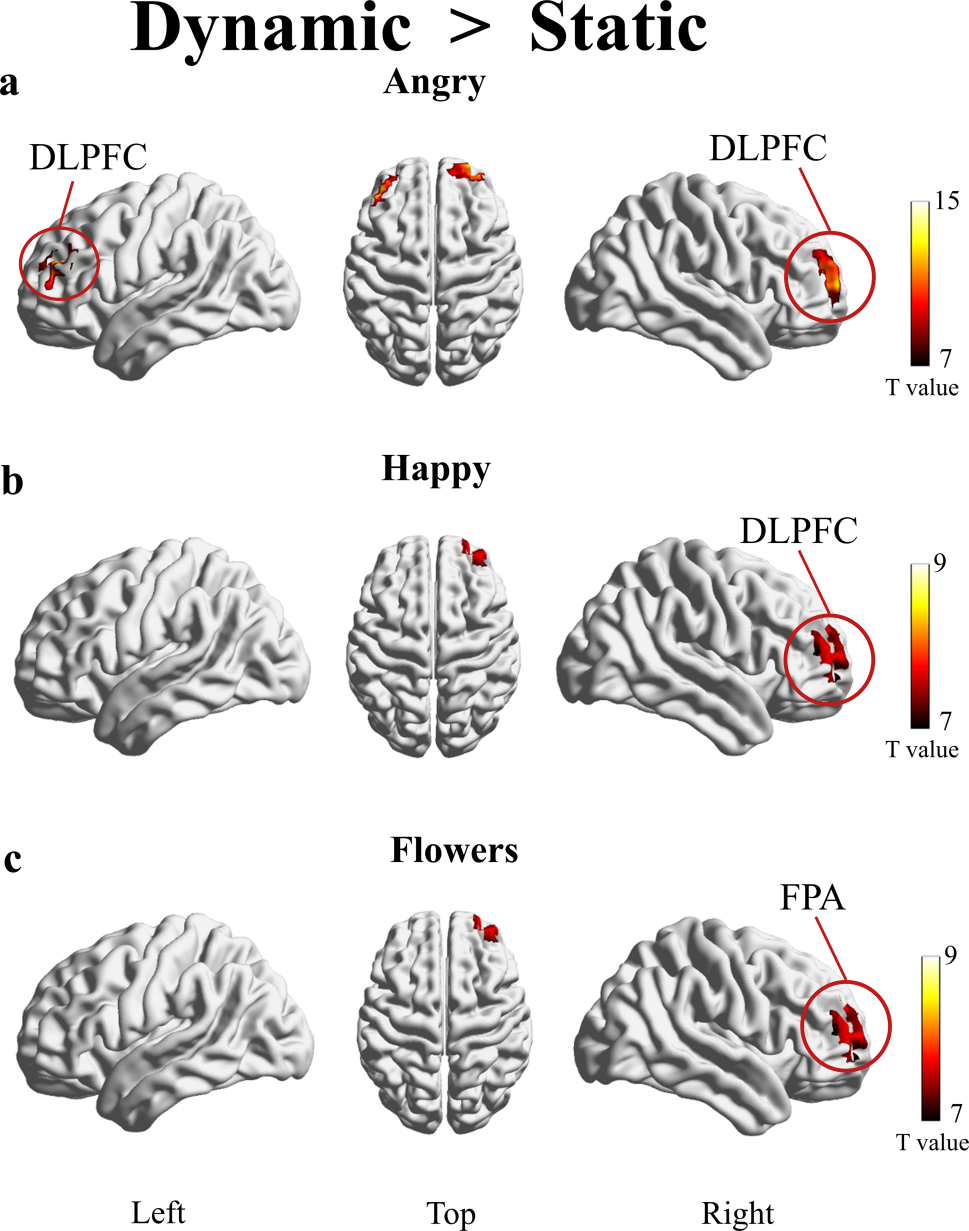
Cortical activation patterns in ASD children during dynamic vs. static emotional face processing (dynamic > static, *p*<0.05, FEWR-corrected for multiple comparisons). **(A)** Dynamic angry faces induced significantly enhanced bilateral dorsolateral prefrontal cortex (DLPFC) activation than static angry faces. **(B)** Dynamic happy faces activated right DLPFC activation than static happy faces. **(C)** Dynamic neutral flower stimuli elicited increased right frontal pole area (FPA) activation compared to static flower images.

A significant main effect of stimulus modality (dynamic vs. static) was observed on cortical activation patterns, F=7.73, *p*<0.05, simple main effect analysis showed that dynamic presentations elicited significantly higher cortical activation in ASD preschoolers. Bilateral DLPFC activation was significantly enhanced during dynamic angry face processing relative to static conditions (**Figure 5A**, dynamic > static, *p*<0.05, FEWR-corrected for multiple comparisons). Right DLPFC showed higher activation during dynamic happy face processing compared to static stimuli (**Figure 5B**, dynamic > static, *p*<0.05, FEWR-corrected). Significantly increased activation in FPA was observed during dynamic neutral flower presentation versus static images (**Figure 5C**).

**Figure 6:**
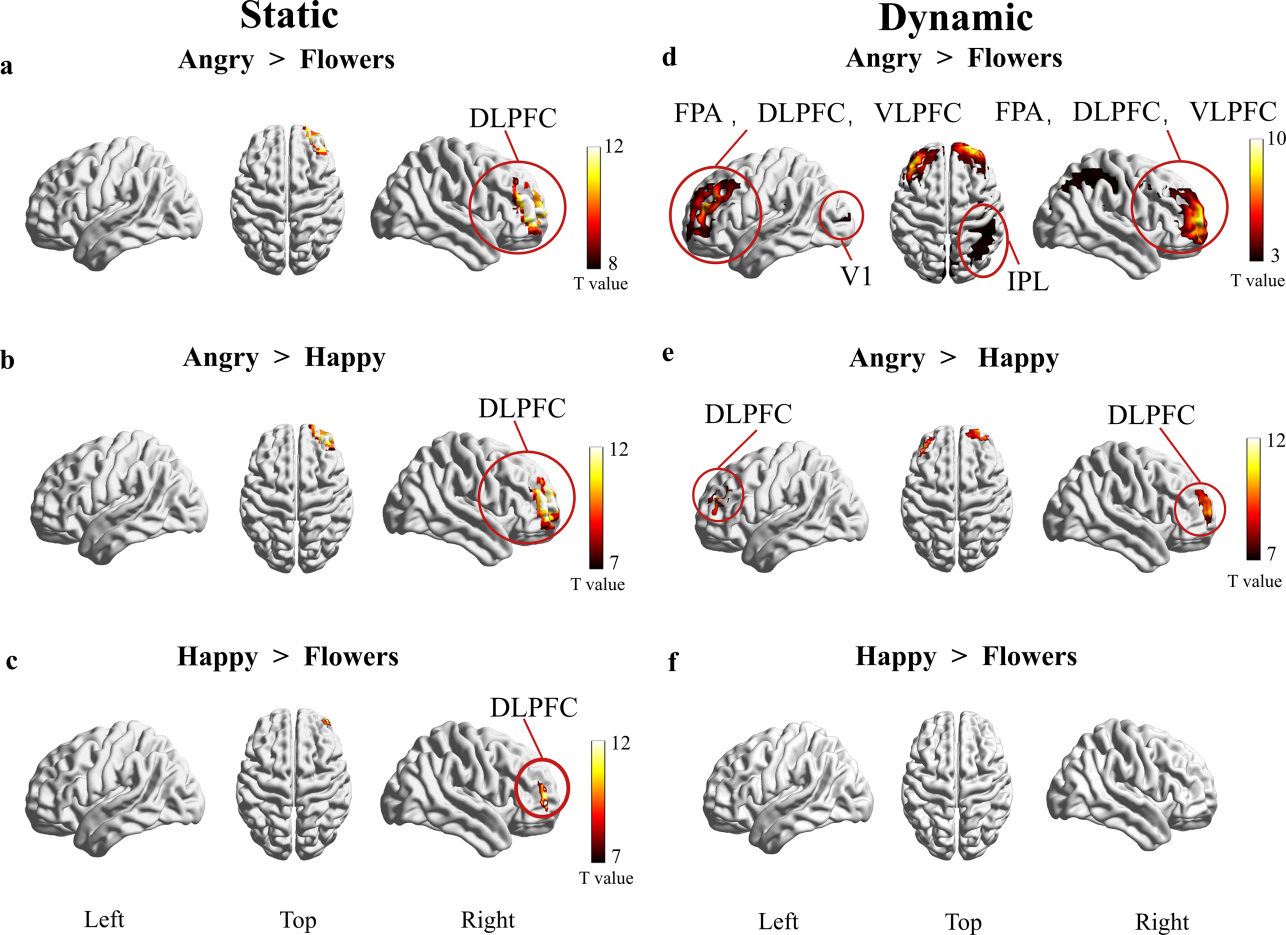
Cortical hemodynamic responses in ASD children to static/dynamic facial stimuli (*p*<0.05, FEWR-corrected for multiple comparisons). **(A)** Static angry faces induced significantly stronger right DLPFC activation than static flower images. **(B)** Static angry faces showed significantly higher activation in the right DLPFC compared to static happy faces. **(C)** Static happy faces also activated the right DLPFC, though with weaker activation than static angry faces. **(D)** Dynamic angry face processing engaged a distributed ROI, including bilateral frontopolar area (FPA), DLPFC, ventrolateral prefrontal cortex (VLPFC), right inferior parietal lobule (IPL), and left primary visual cortex (V1). **(E)** Dynamic angry faces elicited stronger bilateral DLPFC activation than dynamic happy faces. **(F)** No significant cortical activation differences were observed between dynamic happy faces and dynamic neutral flowers.

The two-way ANOVA also revealed that emotional faces (angry, happy and neutral) have a significant effect on cortical activation patters (F=4.65, *p*<0.05),. Cortical activation patterns during emotional face processing is shown in **Figure 6**. Simple main effects analysis showed that in the static facial stimuli conditions, ASD children showed significantly higher activation in right DLPFC during static angry face processing compared to static flower videos (**Figure 6A**). Static angry faces elicited higher right DLPFC activation than static happy faces (**Figure 6B**). Static happy faces also activated the right DLPFC in contrast to static flower neutral flowers (**Figure 6C**). In the dynamic facial stimuli conditions, ASD children showed significantly enhanced activation in bilateral FPA, DLPFC, VLPFC, right inferior parietal lobule (IPL), and left V1 during dynamic angry face processing compared to dynamic flower videos (**Figure 6D**). Dynamic angry face processing elicited significantly stronger bilateral DLPFC activation than dynamic happy faces (**Figure 6E**). No significant differences in cortical activation were observed between dynamic happy faces and neutral flowers (**Figure 6F**).

### Model classification

The training performance of each dataset on different machine learning algorithms, mainly including Naive Bayes, support vector machine (SVM), and deep learning models, is shown in **Table 2**. In the present study, the maximum number of training epochs for each model was 500, with an early-stopping mechanism applied after 20–30 consecutive epochs without improvement on the validation set. The model parameters with the highest accuracy on the validation set were saved and evaluated on the test set. The final classification performance of each model was calculated as the average accuracy across all test sets. The evaluation metrics included accuracy, recall, precision, and F1-score. In terms of the classification performance of the proposed CNN-LSTM model, dynamic facial expression stimuli achieved higher accuracy than static stimuli, with the highest reaching 86.2%.

**Table 2:** Comparison of anger-happiness classification performance across models.

Model	Dynamic				Static			
	Accuracy	Recall	Precision	F1	Accuracy	Recall	Precision	F1
SVM	0.658	0.637	0.599	0.643	0.649	0.637	0.652	0.611
CNN	0.727	0.727	0.726	0.720	0.686	0.684	0.744	0.707
LSTM	0.773	0.773	0.792	0.763	0.721	0.728	0.735	0.721
**CNN+LSTM**	**0.862**	0.827	0.813	0.785	0.636	0.636	0.810	0.534

The bolded values indicate the best performance among all binary classification models.

## Discussion

The aims of this study were to elucidate cortical hemodynamic dynamics during emotional facial processing in preschool children with ASD by fNIRS and to develop a deep learning framework for automatic emotional recognition based on hemodynamic signals. The main findings of the present study were: 1) fNIRS analysis revealed that dynamic facial stimuli elicited significantly greater activation in the prefrontal cortex compared to static stimuli. 2) Angry expressions elicited stronger neural responses than happy expressions. 3) The proposed CNN-LSTM model achieved the highest accuracy (86.2%) in dynamic emotion binary classification, significantly outperforming traditional machine learning methods.

Dynamic stimuli showed significantly higher activation in DLPFC or FPA than static stimuli in the present study. This modality effect aligns with previous studies that dynamic facial cues promote more integrated emotional processing in ASD across childhood and adulthood (17). In the field of neuroimaging, numerous studies have revealed different response patterns to dynamic and static facial emotional stimuli, with dynamic stimuli inducing stronger functional activity in the prefrontal and visual cortices (23). Given that facial expressions are typically dynamic in natural settings, these findings suggest that dynamic representations may be more realistic and more effective in stimulating neuronal activity. The present findings extend prior fMRI research by providing the fNIRS evidence of this dynamic stimulus advantage in preschool-aged children with ASD.

Across both static and dynamic conditions, DLPFC activation was significantly higher for angry versus happy faces. This indicates that young children with ASD exhibit more pronounced hemodynamic responses to anger, aligning with prior research showing heightened neural sensitivity to negative expressions in ASD populations (24–26). Vandewouw et al. (17) used fMRI to demonstrate that compared with neutral flower stimuli, ASD adolescents showed enhanced frontal and occipital activation during dynamic processing of angry/happy faces, consistent with our fNIRS findings (17). DLPFC mediates top-down inhibition of negative emotional responses by regulating amygdala activity (27). For anger, this heightened activation may reflect the emotion’s strong salience, which preferentially captures attentional resources and engages distributed neural networks to process perceived social threats in children with ASD (28, 29).

Under dynamic emotional stimuli, viewing dynamic angry expressions compared with neutral flower videos induced significant activation in multiple brain regions in ASD children, including the bilateral FPA, DLPFC, VLPFC, right IPL, and left V1 (**Figure 6D**). When processing facial stimuli, studies have found that the FPA is more activated than non-facial stimuli, indicating that the FPA is an important part of the facial emotion processing network, participating in emotion generation and regulation (30). Previous research has shown that FPA plays a key role in tracking dynamically changing emotions (31), which is consistent with the FPA activation observed in this study under dynamic stimuli. Leung et al. (32) used magnetoencephalography to reveal that ASD participants showed higher activation levels in the inferior frontal gyrus (including FPA), anterior cingulate cortex, supramarginal and angular gyri, and superior and middle frontal regions when processing angry faces compared with neutral faces (R. C. 32). The IPL, part of the attention network, may reflect visuospatial processing in ASD children (30). Left occipital V1 activation suggests enhanced visual processing of salient dynamic angry stimuli, consistent with greater neural responses to negative versus positive expressions (33).

Previous studies using an Xception CNN-based facial emotion recognition system for high-functioning ASD adults reported a training accuracy of 71% (34). The proposed CNN-LSTM model constructed in this study demonstrated advantages in emotion classification tasks, achieving an accuracy of 86.2% in dynamic facial expression classification (anger vs. happy). LSTMs are well-characterized in the literature for their proficiency in capturing temporal variability within sequential data, a critical capability for fNIRS analysis, as hemodynamic responses to sensory stimuli inherently exhibit time-varying dynamics (15). Specifically, the bidirectional LSTMs employed in the present study can capture bidirectional temporal dependencies, capturing differences in hemodynamic changes during emotional responses. During the training process, the CNN-LSTM model adopts a phased parameter optimization strategy: the CNN part prioritizes learning spatial invariance features, and the LSTM part focuses on temporal pattern mining. The cascaded CNN-LSTM architecture preserves the spatial feature extraction capability of convolutional operations while leveraging recurrent networks to model long-range temporal dependencies. Moreover, compared to pure LSTM networks that are sensitive to noise and may misclassify noise as temporal features, the local aggregation and max-pooling mechanisms of 1DCNN can filter random noise in fNIRS, improving feature robustness.

The combination of CNN and LSTM significantly enhances classification robustness in complex emotional states, aligning with neuroimaging evidence that dynamic stimuli elicit more extensive cortical activation networks in ASD children. This convergence between model performance and neural mechanisms provides empirical support for using dynamic stimuli to optimize feature extraction in neuroimaging-based emotion recognition. Notably, the model’s ability to distinguish between angry and happy expressions, particularly under dynamic stimulus conditions, corresponds to our observation of enhanced activation in the prefrontal-parietal-visual brain regions in response to dynamic angry faces. This linkage between “classification performance” and “neural activation patterns” facilitates the identification of which emotional processing domains are more vulnerable in children with ASD, providing insights to guide the design of targeted interventions, for example, prioritizing threat emotion regulation training for ASD children who exhibit exaggerated neural responses to angry stimuli.

### Limitations

This study has several limitations. First, the present findings center on “ASD-specific internal response characteristics” that inform the development of machine learning frameworks for emotion recognition, rather than advancing definitive assertions regarding “unique neural responses relative to neurotypical populations.” Accordingly, we emphasize the necessity of follow-up studies incorporating neurotypical control groups to validate the clinical utility of our emotion recognition models and clarify their specificity to ASD, this validation step is critical for enhancing the reliability of objective assessment instruments for early intervention in ASD. Second, the sample included only preschoolers with high-functioning ASD, which means the findings may not generalize to children with low-functioning ASD or other age groups. Third, the emotional stimuli were limited to “happy” (positive) and “angry” (negative) expressions, excluding other emotions like sadness or fear, this narrow focus restricts our understanding of the full range of emotional processing in this population.

## Conclusions

This study integrates functional near-infrared spectroscopy (fNIRS) and deep learning to characterize hemodynamic responses during emotional face processing in preschool children with ASD under dynamic and static facial stimuli. Dynamic facial expressions elicited significantly higher prefrontal cortex activation than static stimuli. This aligns with neuroimaging evidence of dynamic stimuli engaging extensive cortical activations in ASD. In particular, compared to neural flower, dynamic angry faces promoted coordinated activation across prefrontal-parietal-visual regions, which may indicate an enhanced neutral processing of social threat cues. The proposed CNN-LSTM model achieved 86.2% accuracy in dynamic anger/happiness classification, outperforming traditional methods by integrating spatial and temporal fNIRS features. These findings validate the utility of dynamic facial stimuli for probing emotional processing in ASD and demonstrate the potential of deep learning for fNIRS-based emotion recognition, which may provide new directions for neurophysiology-informed intervention design. current findings should be interpreted with caution due to the absence of controls, and outline future research plans to recruit matched neurotypical groups for direct comparisons to validate the developed assessment tools.

We note that the current findings should be interpreted with caution due to the absence of a neurotypical control group. Accordingly, we outline future research directions that involve recruiting well-matched neurotypical control groups to conduct direct comparative analyses, with the goal of validating the clinical utility of the developed fNIRS-deep learning assessment tools.

## Data Availability

The raw data supporting the conclusions of this article will be made available by the authors, without undue reservation.
